# Efficient occlusion of oil droplets within calcite crystals†
†Electronic supplementary information (ESI) available: Additional experimental and characterization data, LumiSizer droplet size distributions, SEM images, Raman spectra, XRD, Tables and TGA curves. See DOI: 10.1039/c9sc03372f


**DOI:** 10.1039/c9sc03372f

**Published:** 2019-08-09

**Authors:** Yin Ning, Fiona C. Meldrum, Steven P. Armes

**Affiliations:** a Department of Chemistry , University of Sheffield , Brook Hill , Sheffield , South Yorkshire S3 7HF , UK . Email: Y.Ning@sheffield.ac.uk ; Email: s.p.armes@sheffield.ac.uk; b School of Chemistry , University of Leeds , Woodhouse Lane , Leeds , LS2 9JT , UK

## Abstract

It is well known that oil and water do not mix. Similarly, the incorporation of oil droplets within inorganic crystals is highly counter-intuitive because there is a large difference in surface energy for these two components. Nevertheless, herein we demonstrate the efficient occlusion of ∼250–500 nm oil droplets within 20–40 μm calcite crystals. These droplets are stabilized using various amphiphilic poly(methacrylic acid)–poly(*n*-alkyl methacrylate) diblock copolymer emulsifiers. Both copolymer concentration and diblock compositions affect the extent of occlusion, with optimized conditions producing calcite crystals containing up to 11% oil by mass. Moreover, compressive forces exerted by the growing crystals cause significant deformation of the oil droplets during occlusion. In principle, this protocol enables the incorporation of water-insoluble dyes or hydrophobic nanoparticles within calcite, which is a cheap, naturally-occurring and environmentally-benign mineral. The single crystal nature of this host lattice ensures efficient retention of such guests, while lowering the solution pH leads to triggered release *via* acid dissolution.

## Abstract 2

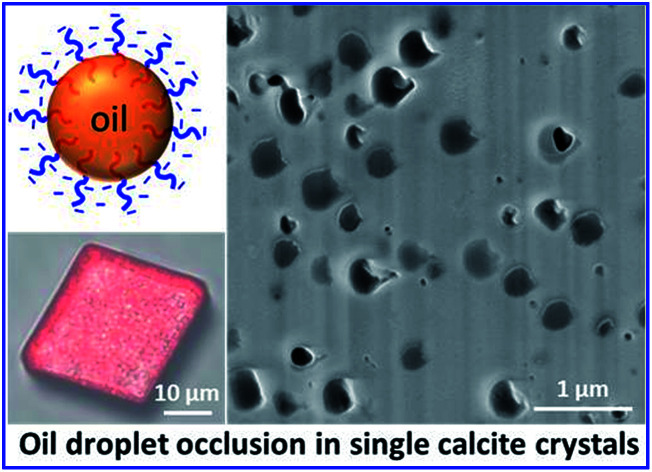
Remarkably efficient occlusion of 250–500 nm oil droplets within single calcite crystals is achieved using anionic amphiphilic diblock copolymer emulsifiers.

## Introduction

Biominerals such as seashells are nanocomposite materials that are composed of organic molecules and mineral hosts and typically exhibit superior mechanical properties compared to their geological counterparts.^1^–^3^ Over the past decade, a range of synthetic protocols have been developed for the incorporation of various guest species (*e.g.* nanoparticles^4^–^9^ or organic molecules^10^–^14^) within host crystals. In principle, efficient additive occlusion should enable the preparation of a range of new nanocomposite crystals^15^–^22^ and, at least in some cases, also enhance our understanding of biomineralization.^23^–^27^ However, the incorporation of *oil droplets* into host crystals has not yet been explored. This is perhaps because, given the marked difference in surface energy between such components, successful occlusion seems at first sight to be extremely unlikely.

Reversible addition–fragmentation chain transfer (RAFT) polymerization offers a versatile route to near-monodisperse functional diblock copolymers.^28^ Moreover, polymerization-induced self-assembly (PISA) enables such diblock copolymers to be conveniently prepared in the form of sterically-stabilized nanoparticles.^29^–^38^ In principle, such amphiphilic diblock copolymers can act as polymeric surfactants (or emulsifiers) for the preparation of nano-sized oil droplets in aqueous media.^39^–^46^

**Scheme 1:**
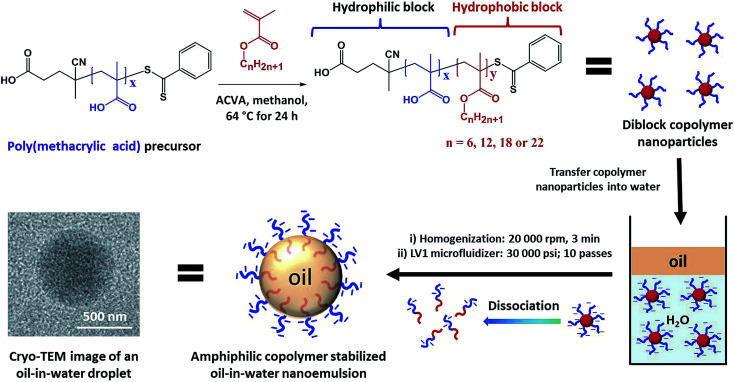
Preparation of diblock copolymer nanoparticles and nanoemulsions. Synthesis of a series of poly(methacrylic acid)–poly(*n*-alkyl methacrylate) diblock copolymer nanoparticles *via* RAFT dispersion polymerization of various *n*-alkyl methacrylates in methanol at 64 °C for 24 h. The schematic cartoon illustrates the preparation of copolymer-stabilized oil-in-water nanoemulsions by (i) high-shear homogenization and (ii) high-pressure microfluidization using a commercial LV1 Microfluidizer. [N.B. the cryo-TEM image obtained for an individual methyl myristate nanoemulsion droplet is consistent with *in situ* dissociation of the anionic copolymer nanoparticles during high pressure microfluidization to form strongly amphiphilic diblock copolymer chains, which then act as a polymeric surfactant].

Herein, a series of new highly amphiphilic diblock copolymers is prepared that not only stabilizes such oil droplets but also confers appropriate surface functionality to ensure their efficient occlusion with single calcite (CaCO_3_) crystals. First, poly(methacrylic acid)_*x*_–poly(lauryl methacrylate)_*y*_ nanoparticles were prepared by RAFT dispersion polymerization of lauryl methacrylate in methanol (Scheme 1). For brevity, poly(methacrylic acid)_*x*_–poly(lauryl methacrylate)_*y*_ copolymers are denoted as PMAA_*x*_–PLMA_*y*_, where *x* denotes the mean degree of polymerization (DP) of the anionic block and *y* indicates the variable DP of the hydrophobic block. This amphiphilic copolymer emulsifier was subsequently used to stabilize oil-in-water nanoemulsions, with the PMAA chains being located in the aqueous phase while the PLMA block resides within the oil droplets (Scheme 1). The copolymer concentration and diblock composition were systematically varied to evaluate their influence on the extent of oil droplet occlusion within growing CaCO_3_ crystals.

## Results and discussion

### Preparation and characterization of copolymer emulsifiers and nanoemulsions

Gel permeation chromatography (GPC) studies confirmed that both the PMAA_*x*_ precursor and the PMAA_*x*_–PLMA_*y*_ diblock copolymers exhibited narrow molecular weight distributions (*M*_w_/*M*_n_ < 1.30; Tables S1 and S2†), indicating good RAFT control. The PMAA_*x*_–PLMA_*y*_ nanoparticles were transferred into aqueous media by dialysis against water before being employed as a copolymer emulsifier for the stabilization of oil-in-water emulsions (Scheme 1). Food-grade methyl myristate was used as the model oil for the preparation of oil-in-water nanoemulsions and the oil/water volume ratio was fixed at 0.10. Typically, methyl myristate (0.50 mL) containing Nile Red dye (0.25 mg g^–1^) was added to 5.0 mL of an aqueous dispersion containing 0.10–0.80% w/w PMAA_*x*_–PLMA_*y*_ nanoparticles. First, a coarse polydisperse pre-emulsion (10–200 μm diameter) was prepared by high-shear homogenization. Such droplets were then passed ten times through an LV1 Microfluidizer at 30 000 psi to produce emulsions with mean oil droplet diameters of ∼250–570 nm (see the cryo-TEM image recorded for an individual methyl myristate nanoemulsion droplet in Scheme 1).

**Fig. 1:**
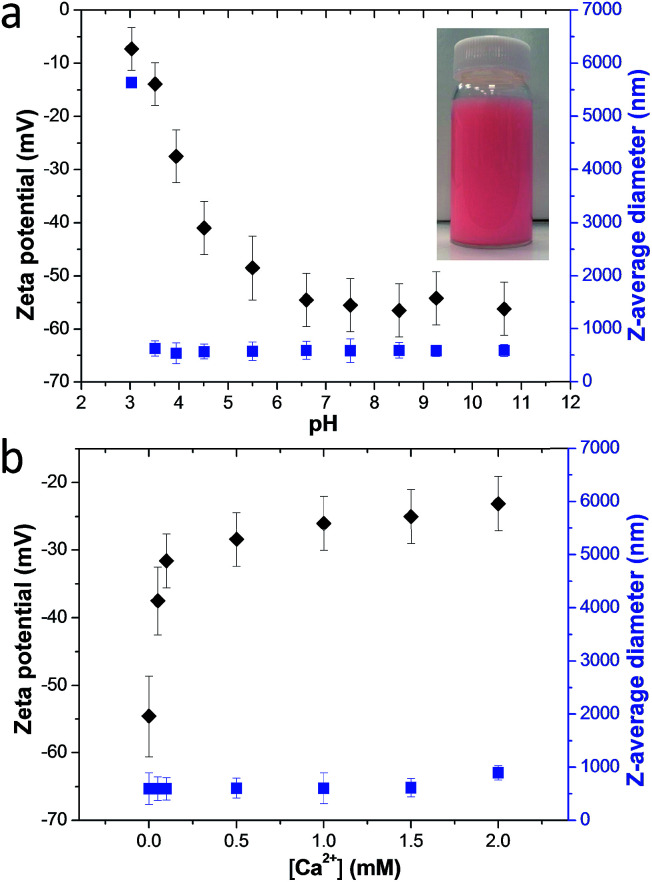
Dynamic light scattering and aqueous electrophoresis data obtained for a PMAA_156_–PLMA_80_ stabilized methyl myristate-in-water nanoemulsion. (a) Variation of zeta potential and hydrodynamic droplet diameter with pH (inset: digital photograph of the nanoemulsion, with the oil phase containing 0.25 mg g^–1^ Nile Red dye). (b) Variation of zeta potential and hydrodynamic droplet diameter with [Ca^2+^] at pH 9.

Calcium carbonate crystals were grown using the ammonia diffusion method.^47^ This protocol involves the diffusion of ammonia and carbon dioxide into water to form an alkaline aqueous solution (pH ∼ 9) in the presence of 1.5 mM CaCl_2_. Methyl myristate-in-water nanoemulsions prepared using PMAA_156_–PLMA_80_ exhibited a zeta potential of ∼–54 mV above pH 6: this is because the PMAA stabilizer chains located at the nanoemulsion droplet surface become ionized, leading to highly anionic character (Fig. 1a).^48^ This is important, because surface anionic carboxylate groups play a key role in dictating additive occlusion within calcite.^25^ On introducing Ca^2+^ ions at pH 9, the zeta potential of this nanoemulsion became progressively less negative (*e.g.* ∼–25 mV at 1.5 mM CaCl_2_, Fig. 1b), which suggests Ca^2+^ binding to the PMAA chains.^49^ However, overall anionic character is retained, which is essential for nanoparticle occlusion.^50^ It is important to establish that the nanoemulsion droplets remain stable on the time scale of this reaction (24 h). The *z*-average diameter of the nanoemulsion droplets is relatively constant at pH 9 in the presence of Ca^2+^ ions as determined by dynamic light scattering (DLS) (Fig. 1). These observations suggest that the nanoemulsion should remain stable during the occlusion experiments (pH 9; Ca^2+^ concentration: 0–1.5 mM), which is a prerequisite for their incorporation within the host crystal.

### Effect of varying the copolymer concentration and composition on the extent of occlusion

**Fig. 2:**
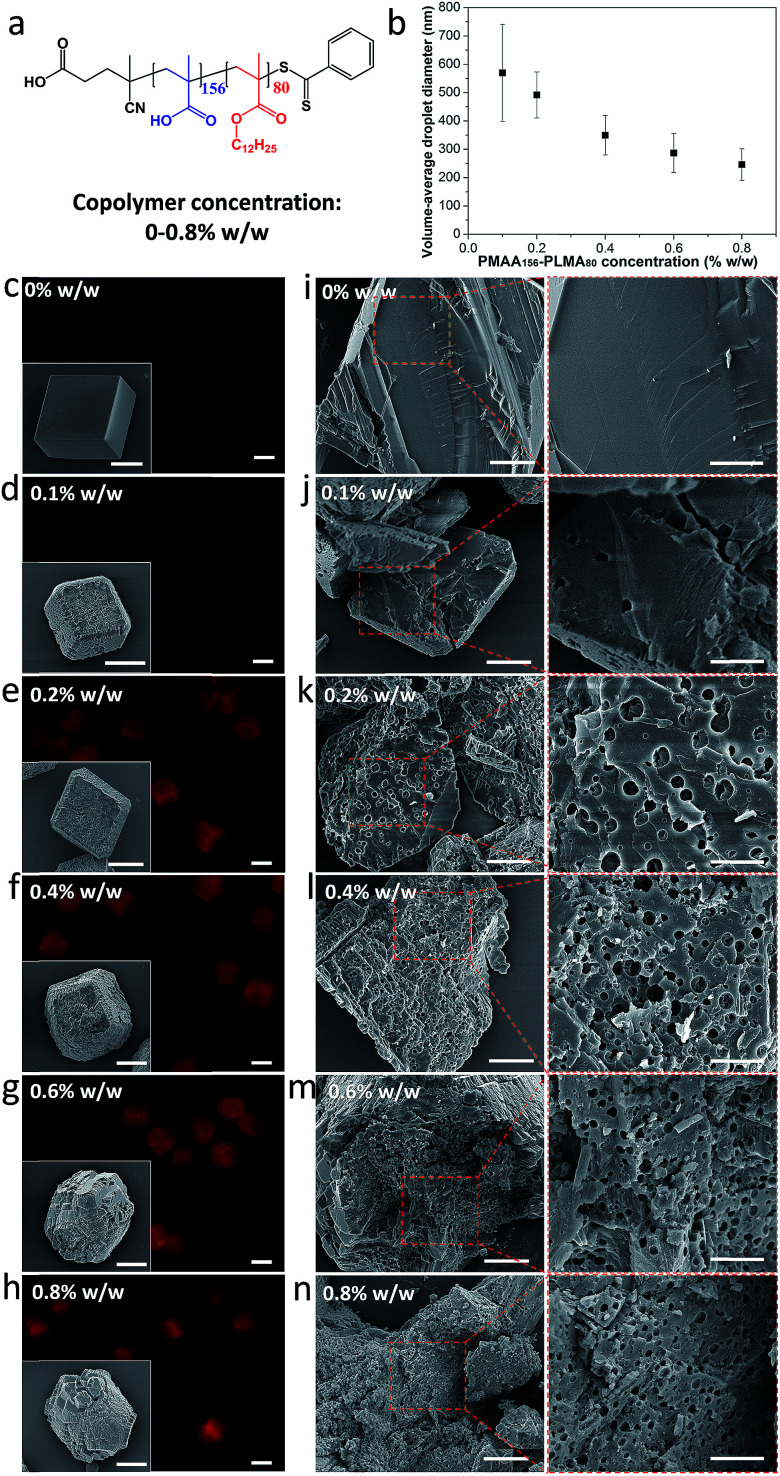
Effect of varying the concentration of PMAA_156_–PLMA_80_ emulsifier on the extent of occlusion of nanoemulsion droplets within calcite. (a) Chemical structure of PMAA_156_–PLMA_80_ diblock copolymer; (b) reduction in the mean droplet diameter of the methyl myristate-in-water nanoemulsion on increasing the PMAA_156_–PLMA_80_ concentration. (c–n) Calcite crystals precipitated in the presence of various nanoemulsions stabilized using PMAA_156_–PLMA_80_ concentrations up to 0.80% w/w. (c–h) Fluorescence microscopy images and corresponding SEM images (see insets) illustrating the surface morphology of the intact calcite crystals. To aid comparison between images, only those crystals that lie relatively flat are shown here. Further SEM images are provided in Fig. S3.† (i–n) SEM images revealing the internal morphology of randomly-fractured calcite crystals. Scale bars for the fluorescence microscopy images, inset SEM images, low magnification SEM images and high magnification SEM images are 20 μm, 10 μm, 5 μm and 2 μm, respectively.

The volume-average droplet diameter of a methyl myristate-in-water nanoemulsion prepared using a PMAA_156_–PLMA_80_ emulsifier (Fig. 2a) is systematically reduced from 570 nm to 246 nm on increasing the copolymer concentration from 0.10 to 0.80% w/w (Fig. 2b), as determined *via* analytical photocentrifugation (Fig. S1†). Calcite was precipitated at a nanoemulsion concentration of 0.10% v/v, because higher concentrations led to formation of ill-defined crystals (Fig. S2†). In the absence of any additives, well-defined rhombohedral calcite crystals with smooth surfaces were obtained (Fig. 2c, inset). In contrast, calcite crystals precipitated in the presence of the nanoemulsions had rough surfaces (Fig. 2d–h, insets). Moreover, higher concentrations of PMAA_156_–PLMA_80_ emulsifier afforded crystals that were elongated in the [001] direction (Fig. 2g–h, insets). This is because higher emulsifier concentrations produce a larger number of finer oil droplets (Fig. 2a). This in turn leads to a significant change in the crystal morphology.^51^ In addition, we have previously reported that smaller organic nanoparticles affect the crystallization habit and thus change the crystal morphology, presumably because it is easier for them to adsorb at the growing steps and hence interact with the growing crystals.^49^,^51^ To avoid any significant changes in the CaCO_3_ morphology (Fig. 2f–h), a fixed *molar* copolymer concentration (2.94 × 10^–7^ M; corresponding to 0.20% w/w PMAA_156_–PLMA_80_) was used in the following experiments.

To investigate the extent of nanoemulsion occlusion within these calcite crystals, the internal structure of randomly-fractured crystals was examined by scanning electron microscopy (SEM). Methyl myristate evaporates under the ultrahigh vacuum conditions required for SEM studies, so voids are observed instead of the original oil droplets. In control experiments, the internal structure of fractured pure calcite crystals was featureless (Fig. 2i) and such crystals were non-fluorescent (Fig. 2c). Minimal occlusion was observed for calcite prepared in the presence of the largest nanoemulsion (volume-average diameter = 570 ± 171 nm) prepared using 0.10% w/w PMAA_156_–PLMA_80_ (Fig. 2j). This is consistent with the weak fluorescence observed for such crystals (Fig. 2d). However, uniform, dense occlusion was observed for calcite crystals prepared in the presence of finer nanoemulsions (volume-average diameter = 492 ± 81 nm to 246 ± 56 nm) prepared using higher emulsifier concentrations (Fig. 2k–n). Moreover, highly fluorescent crystals containing smaller voids were observed under such conditions. The void dimensions were comparable to those of the corresponding nanoemulsion in each case.

**Fig. 3:**
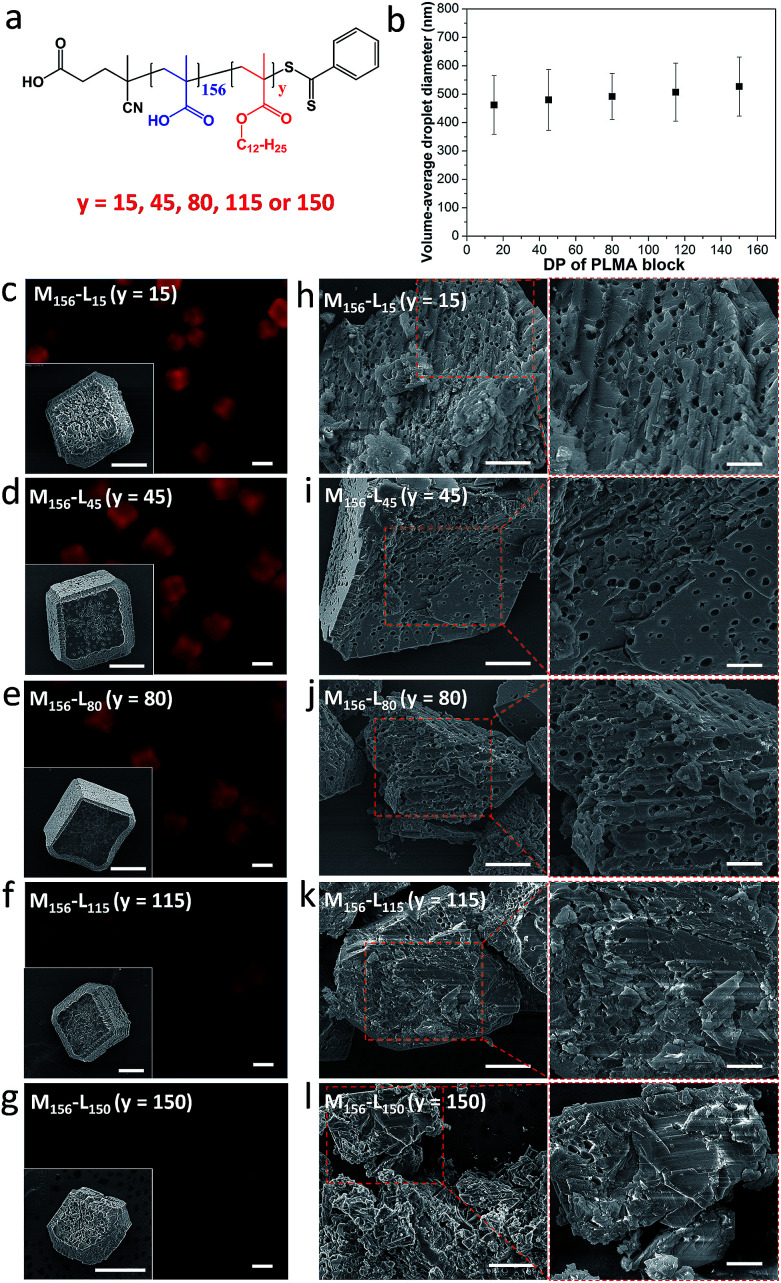
Effect of varying the PMAA_156_–PLMA_*y*_ diblock copolymer composition on the extent of occlusion of oil droplets within calcite. (a) Chemical structure for a series of PMAA_156_–PLMA_*y*_ diblock copolymers; (b) mean droplet diameter of the methyl myristate-in-water nanoemulsions *vs.* the PLMA block DP (*y* value) for the PMAA_156_–PLMA_*y*_ diblock copolymer emulsifier. (c–l) Calcite crystals prepared in the presence of various nanoemulsions stabilized using the same *molar* concentration of PMAA_156_–PLMA_*y*_ diblock copolymer (2.94 × 10^–7^ M) where 15 ≤ *y* ≤ 150. (c–g) Fluorescence microscopy images and corresponding SEM images (see insets) illustrating the surface morphology of the intact calcite crystals. More SEM images can be found in Fig. S3.† (h–l) SEM images revealing the internal morphology of randomly-fractured calcite crystals. Scale bars for the fluorescence microscopy images, inset SEM images, low magnification SEM images and high magnification SEM images are 20 μm, 10 μm, 5 μm and 2 μm, respectively.

In a second series of experiments, the amphiphilic character of the diblock copolymer emulsifier was tuned by systematically varying the mean PLMA block DP (*y*) from 15 to 150 (Fig. 3a). At a fixed *molar* copolymer concentration of 2.94 × 10^–7^ M (which corresponds to 0.10–0.30% w/w), stable nanoemulsions could be prepared regardless of the diblock composition, but employing longer PLMA blocks led to slightly larger nanoemulsion droplets (Fig. 3b). Rhombohedral crystals with rough surfaces were obtained in the presence of a series of 0.10% v/v nanoemulsions stabilized using various diblock copolymers (Fig. 3c–g, insets). Uniform occlusion was observed for PMAA_156_–PLMA_*y*_ emulsifiers with *y* values of 15, 45 or 80 (Fig. 3h–j). In contrast, significantly lower levels of occlusion were achieved for *y* = 115 or 150 (Fig. 3k and l). These observations are consistent with the corresponding fluorescence images for such crystals (Fig. 3c–g).

In principle, diblock copolymer nanoparticles comprising longer hydrophobic blocks should be less prone to *in situ* dissociation during high-pressure microfluidization. To avoid this problem, we fixed the hydrophobic block at a relatively short DP of approximately 45 and varied the length of the pendent alkyl group by using different *n*-alkyl methacrylate monomers (Scheme 1 and Fig. S4a†). Stable nanoemulsions were obtained for all of these diblock copolymer emulsifiers (Fig. S4b†). However, both fluorescence microscopy and SEM studies indicated only rather low levels of occlusion for nanoemulsions stabilized by poly(methacrylic acid)–poly(behenyl methacrylate) (PMAA_156_–PBeMA_45_) (Fig. S4f and j†). Although the hydrophobic block DP is fixed, its volume fraction increases when using larger pendent *n*-alkyl groups. This should lead to less efficient packing of the copolymer chains at the oil/water interface. This hypothesis is consistent with the smaller mean diameter observed for nanoemulsions stabilized using the PMAA_156_–PBeMA_45_ copolymer (see Fig. S4b†).

In view of the above studies, the effect of varying the DP of the PMAA block on the preparation of nanoemulsions and their subsequent occlusion was also investigated. Thus we fixed the PLMA block DP at ∼45 and prepared a series of PMAA_*x*_–PLMA_∼45_ copolymers with *x* = 40, 82 or 156 (see Fig. S5a†). The nanoemulsion droplet diameter was systematically reduced on increasing the PMAA block DP (Fig. S5b†). Calcite crystals prepared in the presence of nanoemulsions stabilized by PMAA_40_–PLMA_42_ and PMAA_82_–PLMA_42_ exhibited a well-defined rhombohedral morphology, while truncated edges were observed when using PMAA_156_–PLMA_45_-stabilized nanoemulsions (Fig. S5c–e,† insets). This suggests that a longer PMAA block facilitates stronger interaction between the nanoemulsion droplets and the calcite lattice. Indeed, SEM studies suggested that only minimal occlusion occurs when using PMAA_40_–PLMA_42_-stabilized nanoemulsions, whereas somewhat higher occlusion was obtained for PMAA_82_–PLMA_42_-stabilized nanoemulsions (Fig. S5d and g†). Thus, a sufficiently long enough PMAA block appears to be essential for efficient occlusion of nanoemulsion droplets within calcite.^25^

Raman microscopy (Fig. S6†) and powder XRD studies (Fig. S7†) of the crystals prepared in these experiments confirmed their expected structure. Pure calcite was obtained when using 0.20% w/w PMAA_156_–PLMA_80_ emulsifiers. However, using a relatively high PMAA_156_–PLMA_80_ copolymer concentration (0.80% w/w) led to predominantly calcite with a small amount of vaterite (Fig. S7†). The extent of occlusion was determined by thermogravimetry (Fig. S8†). Using 0.40% w/w PMAA_156_–PLMA_80_ produced calcite crystals containing up to 11.8% oil by mass (Fig. S9a†). However, higher copolymer concentrations only led to changes in the calcite morphology, rather than higher degrees of occlusion (Fig. S9a† and 2f–h insets). At a fixed *molar* copolymer concentration, using a relatively short hydrophobic PLMA block (*i.e.* DP = 15, 45 or 80) led to greater occlusion (Fig. S9b†). On the other hand, hydrophobic blocks containing relatively long pendent alkyl groups (*e.g.* PBeMA) reduced occlusion levels significantly (Fig. S9c†). Moreover, a sufficiently long PMAA block (*e.g.* DP = 156) is required for efficient nanoemulsion occlusion within calcite crystals. These thermogravimetry data are consistent with the corresponding SEM observations. Given that crystallization normally favors impurity expulsion rather than occlusion,^52^,^53^ it is remarkable that oil droplets of up to 500 nm diameter can be occluded within calcite with such high efficiency.

### Morphological deformation of the occluded oil droplets

**Fig. 4:**
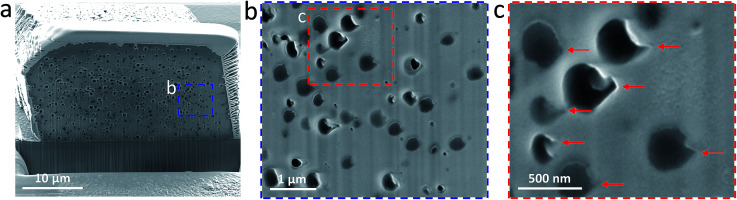
Internal structure of oil droplets occluded within calcite crystals. (a) Low magnification SEM image showing a cross-section parallel to (104) face obtained after focused ion beam (FIB) milling; (b and c) show high magnification SEM images recorded for the areas indicated in (a and b), respectively; the red arrows in (c) indicate the non-isotropic voids and their distinctive tips.

Crystal etching *via* focused ion beam (FIB) milling allowed examination of cross-sections parallel to the (104) face. Such experiments confirm that voids are distributed throughout the whole crystal (Fig. 4). Interestingly, these cavities do not possess the spherical morphology of the original oil droplets, indicating that the oil droplets undergo deformation during occlusion.^49^,^52^ It is worth noting that in the previous studies the copolymer micelles^49^,^52^ or vesicles^25^ became somewhat elongated after their occlusion. However, the cavities formed in this study are rather asymmetric, suggesting that the growing crystal exerts anisotropic compression forces on the oil droplets. These liquid droplets are much more deformable compared to the diblock copolymer micelles or vesicles, thus a more significant change in morphology is observed during their occlusion. Inspection of these cavities located at the outer surface reveal ‘tips’ pointing toward the (104) face (Fig. 4c, red arrows). We hypothesize that oil droplets initially adsorb onto the growing crystal faces *via* their anionic PMAA chains, which contain strongly-bound Ca^2+^ cations. As the crystal step advances, these pinned oil droplets are subjected to compressive forces, which lead to formation of a ‘tip’ in the final stages of occlusion.

### Release of cargo-loaded oil droplets from the host calcite crystals

**Fig. 5:**
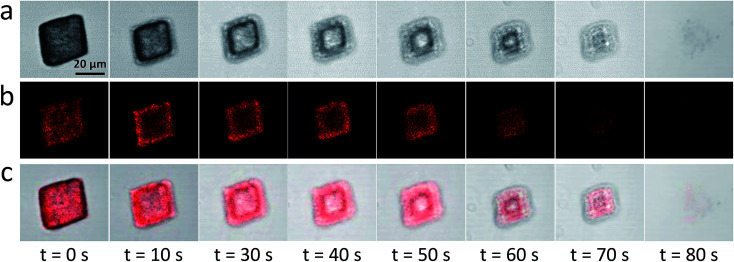
*In situ* monitoring of the release of oil droplets from calcite crystals on addition of simulated gastric fluid (SGF, pH 1.2) by confocal laser scanning microscopy (CLSM). Such calcite crystals are occluded with up to 11 wt% methyl myristate droplets (dyed with Nile Red and stabilized using 2.94 × 10^–7^ M PMAA_156_–PLMA_45_ diblock copolymer): (a) bright-field images; (b) CLSM images; (c) merged images. These images were recorded at various time points at the same magnification [see scale bar shown in (a)].

Occlusion of other oil droplets (*e.g.* sunflower oil, multi-component fragrances, iso-hexadecane, *etc*) within calcite can also be achieved using essentially the same protocol (Fig. S10†). Moreover, gold or magnetite nanoparticles dispersed in iso-hexadecane droplets can be incorporated within calcite (Fig. S10†). Such proof-of-concept experiments demonstrate that new classes of functional nanocomposite materials can be designed. Calcite is an abundant, biocompatible and pH-sensitive mineral. In principle, it could be used as an ideal inert matrix for gastric delivery of many hydrophobic drugs (*e.g.* sucralfate, doxorubicin).^53^,^54^ Acid-triggered release experiments using oil-loaded calcite crystals were performed to examine this hypothesis. Thus, acid dissolution of calcite containing occluded Nile Red-loaded oil droplets was monitored using confocal laser scanning microscopy (CLSM). The initial crystal is uniformly fluorescent (*t* = 0, Fig. 5), indicating uniform distribution of the oil droplets within the calcite. On addition of simulated gastric fluid (SGF, pH 1.2), the outer surface of the oil-loaded calcite crystal begins to dissolve first (*t* = 10 s); such surface erosion leads to the formation of the unusual edge effect shown in Fig. 5a. Thus, pH-triggered release of drug-loaded oil droplets can be achieved for targeted gastric delivery.^55^ Moreover, the poly(methacrylic acid) stabilized drug-loaded oil droplets undergo coalescence below pH 3 (Fig. 1a), which should aid localized release at a solution pH of 3 (or lower). Crystal dissolution aslo occurs at pH 4, but at a much slower rate. It is also worth noting that the occluded oil droplets can be retained within the calcite crystals at up to 400 °C because there is no discernible mass loss at this temperature (see Fig. S8†). This suggests the single crystal nature of calcite should ensure long-term retention of guest species.

## Conclusions

In summary, the occlusion of oil droplets within a crystalline lattice (calcite) is demonstrated for the first time. This is achieved using anionic diblock copolymer emulsifiers to stabilize the nano-sized oil droplets, which are prepared by high-pressure microfluidization. Remarkably, thermogravimetric analyses indicate that the extent of occlusion can be as high as ∼11% oil by mass, under optimized conditions. Furthermore, FIB-SEM studies of calcite crystals indicate that significant deformation of oil droplets occurs during occlusion. This new occlusion protocol enables oil-soluble or oil-dispersable guests (*e.g.* dyes or hydrophobic nanoparticles) to be incorporated within host crystals. Moreover, the single crystal nature of calcite should ensure long-term retention of such small molecules. Finally, the relatively low cost, high biocompatibility and environmentally-benign nature of calcite combined with opportunities for triggered release *via* either mechanical rupture or pH adjustment suggests potential controlled-release applications.

## Experimental

### Materials

Methacrylic acid (MAA), *n*-hexyl methacrylate (HMA), lauryl methacrylate (LMA), stearyl methacrylate (SMA), behenyl methacrylate (BeMA), 4,4′-azobis(4-cyanovaleric acid) (ACVA; 99%), (4-cyano-4-(phenylcarbonothioylthio)pentanoic acid) (CPCP), methyl myristate, sunflower oil, isohexadecane, iron(ii) chloride tetrahydrate, iron(iii) chloride hexahydrate, gold(iii) chloride trihydrate, 1-octadecene, oleylamine, oleic acid, concentrated ammonia solution (35%), ammonium carbonate and calcium chloride hexahydrate were all purchased from Sigma-Aldrich (UK) and used as received. Deionized water was obtained from an in-house Elgastat Option 3A water purification unit. All solvents were obtained from Sigma-Aldrich (UK). Voyager Zen fragrance was supplied by P & G.

### Synthesis of poly(methacrylic acid) (PMAA_*x*_) macro-CTAs (*x* = 40, 82 or 156)

A typical protocol for the synthesis of PMAA_156_ macro-CTA is as follows. A round-bottomed flask was charged with CPCP (162.3 mg; 0.581 mmol), ACVA (32.6 mg; 0.116 mmol; [CPCP]/[ACVA] = 5), ethanol (15 g) and MAA (10.0 g; 116.2 mmol; target DP = 200). The sealed reaction vessel was purged with nitrogen for 30 min and placed in a pre-heated oil bath at 70 °C for 5 h. The resulting crude PMAA macro-CTA was purified by precipitation into a ten-fold excess of diethyl ether (three times). Then this PMAA macro-CTA was dissolved in water and isolated by lyophilization. A mean DP of 156 was calculated for this macro-CTA using ^1^H NMR spectroscopy by comparing the integrated signal intensity assigned to the aromatic protons with that of the methacrylic polymer backbone protons. After exhaustive methylation using trimethylsilyldiazomethane, THF GPC analysis indicated *M*_n_ = 17 400 g mol^–1^ and *M*_w_/*M*_n_ = 1.10 (*vs.* a series of near-monodisperse poly(methyl methacrylate) standards). Lower molecular weight macro-CTAs (*e.g.* PMAA_40_ macro-CTA; *M*_n_ = 4800 g mol^–1^; *M*_w_/*M*_n_ = 1.15 and PMAA_82_ macro-CTA; *M*_n_ = 9200 g mol^–1^; *M*_w_/*M*_n_ = 1.15) were prepared by using the same mass of MAA and reducing the mass of CPCP accordingly (see summary Table S1†).

### Synthesis of poly(methacrylic acid)_*x*_–poly(lauryl methacrylate)_*y*_ (PMAA_*x*_–PLMA_*y*_) diblock copolymer nanoparticles (*x* = 40, 82 or 156; *y* = 15, 45, 80, 115 or 150)

A typical synthesis protocol of PMAA_156_–PLMA_80_*via* RAFT dispersion polymerization of lauryl methacrylate is as follows: PMAA_156_ macro-CTA (500 mg, 36.5 μmol), ACVA (2.0 mg, 7.3 μmol) and methanol (11.2 g) were weighed into a 50 mL flask; thereafter, LMA (742.5 mg, 2.9 mmol) was charged. The vial was sealed and purged by nitrogen for 20 min before being placed into a preheated oil bath at 70 °C for 24 h. The resulting diblock copolymer nanoparticles were dialyzed against deionized water using dialysis tubing with a molecular weight cut-off of 3500 Da.

### Synthesis of poly(methacrylic acid)_*x*_-based diblock copolymer nanoparticles with various hydrophobic core-forming monomers, *e.g.* hexyl methacrylate (HMA), stearyl methacrylate (SMA), behenyl methacrylate (BeMA)

The following synthesis protocol for PMAA_156_–PHMA_45_*via* RAFT dispersion polymerization of *n*-hexyl methacrylate (HMA) is representative. PMAA_156_ macro-CTA (500 mg, 36.5 μmol), ACVA (2.0 mg, 7.3 μmol) and methanol (7.0 g) were weighed into a 50 mL round-bottomed flask; thereafter, HMA (279.5 mg, 1.6 mmol) was charged. The vial was sealed and purged with nitrogen gas for 20 min before being placed into a preheated oil bath at 70 °C for 24 h. The HMA monomer conversion was 93% and the conversion reached 100% for SMA and BeMA, as determined by ^1^H NMR spectroscopy. The resulting diblock copolymer nanoparticles were dialyzed against deionized water using dialysis tubing with a molecular weight cut-off of 3500 Da.

### Preparation of nanoemulsions

A range of oils, including methyl myristate, sunflower oil, isohexadecane, and a multi-component fragrance were selected as the oil phase for the nanoemulsions. In addition, gold nanoparticles dispersed in isohexadecane and magnetite nanoparticles dispersed in isohexadecane were also used as the oil phase. A typical protocol for the preparation of a methyl myristate-in-water nanoemulsion is as follows: 0.50 mL oil was added to 5.0 mL of water containing desired amount of diblock copolymer nanoparticles and homogenized for 3 min at 20 °C using an IKA Ultra-Turrax T-18 homogenizer operating at 20 000 rpm. This relatively coarse precursor emulsion (mean droplet diameter ≫ 1 μm) was further processed using an LV1 low-volume Microfluidizer (Microfluidics, USA). The applied pressure was adjusted to 30 000 psi and the emulsion was passed through the LV1 cell ten times. For fluorescence microscopy and confocal microscopy imaging, Nile Red was added to the methyl myristate (0.25 mg dye per gram of oil).

### Precipitation of calcium carbonate crystals in the presence of nanoemulsions

Occlusion experiments were conducted immediately after preparation of the fresh nanoemulsion. CaCO_3_ crystals were precipitated onto a glass slide placed at the base of an aqueous solution containing 1.5 mM CaCl_2_ and 0.1% vol oil-in-water nanoemulsion (various oils were evaluated in such occlusion experiments) by exposure to ammonium carbonate vapor (2–3 g, placed at the bottom of the dessicator) for 24 h at 20 °C. Then the glass slide was removed from the solution and washed three times with deionized water followed by three rinses with ethanol. Each occlusion experiment was repeated at least twice and consistent results were obtained in each case.

### Release of cargo-loaded oil droplets from the host calcite crystals

Calcite crystals occluded with Nile Red-labeled oil droplets (11% oil by mass) were grown on a glass slide. A few drops of simulated gastric fluid (SGF, pH 1.2) was then placed on this slide and acid dissolution of the crystals was monitored using confocal laser scanning microscopy at 20 °C.

## Characterization

### ^1^H NMR spectroscopy

^1^H NMR spectra were recorded using a Bruker Avance 400 spectrometer operating at 400 MHz using either CD_3_OD or d_6_-DMSO as solvents. The number of scans averaged per spectrum was 64.

### Gel permeation chromatography (GPC)

For THF GPC studies, the carboxylic acid groups on the PMAA block were exhaustively methylated using trimethylsilyldiazomethane. The GPC set-up consisted of two 5 μM mixed C columns connected to a WellChrom K-2301 refractive index detector. The mobile phase was HPLC-grade THF containing 1.0% glacial acetic acid and 0.05% w/v butylhydroxytoluene (BHT) at a flow rate of 1.0 mL min^–1^. Molecular weights were calculated with respect to a series of near-monodisperse poly(methyl methacrylate) standards.

### Dynamic light scattering (DLS)

DLS measurements were conducted using a Malvern Zetasizer NanoZS instrument by detecting back-scattered light at an angle of 173°. Aqueous dispersions of the samples were diluted to 0.1% vol using deionized water. Aqueous electrophoresis measurements were conducted using disposable folded capillary cells supplied by Malvern (DTS1070) using the same instrument with 1 mM NaCl as a background electrolyte. For zeta potential *vs.* pH and zeta potential *vs.* Ca^2+^ concentration studies, the concentration of the nanoemulsion was fixed at 0.10% v/v.

### Scanning electron microscopy (SEM)

Calcite crystal morphologies were examined using a field emission scanning electron microscope (Inspect F instrument). Samples were fractured by placing a clean glass slide on top of the glass slide supporting the calcite crystals, pressing down lightly and twisting one slide relative to the other. Afterwards, glass slides supporting fractured CaCO_3_ crystals were gold-coated (15 mA, 1 min). A relatively low accelerating voltage (5 kV) was applied in order to minimize sample charging. Focused ion beam (FIB) milling studies were performed using a FEI Helios NanoLab G3 UC SEM instrument to prepare cross-sections through individual crystals. FIB milling was conducted using a gallium ion current (initially 21 nA, gradually reduced to 80 pA) at an acceleration voltage of 30 kV. A final polish was conducted using a gallium ion current of 80 pA.

### Cryo-transmission electron microscopy (Cryo-TEM)

Cryo-TEM studies were conducted on a 0.50% v/v methyl myristate-in-water nanoemulsion of 492 ± 81 nm diameter frozen onto holey carbon grids using a FEI Tecnai Arctica instrument operating at 200 kV.

### Fluorescence microscopy and confocal microscopy

Fluorescence images were recorded using a Zeiss Axio Scope A1 microscope fitted with an AxioCam 1Cm1 monochrome camera. Images were captured and processed using ZEN lite 2012 software. Confocal images were recorded using a Nikon A1 confocal microscope equipped with Nikon elements software.

## Conflicts of interest

S. P. A. and Y. N. declare a patent application filed by The University of Sheffield protecting certain aspects of this study.

## Supplementary Material

Supplementary information
