# Supplementary material for: Efficient occlusion of oil droplets within calcite crystals
Source: Chem Sci. 2019 Aug 9;10(39):8964–72. doi: 10.1039/c9sc03372f (PMC7472555; doi:10.1039/c9sc03372f)
Supplement: Supplementary file 1 [file SC-010-C9SC03372F-s001.pdf]

Electronic Supplementary Material (ESI) for Chemical Science.
This journal is © The Royal Society of Chemistry 2019

Supplementary Information for:

Efficient occlusion of oil droplets within calcite crystals

Yin Ning,[*a] Fiona C. Meldrum[b] and Steven P. Armes[*a]

[a] Department of Chemistry, University of Sheffield, Brook Hill, Sheffield, South Yorkshire S3 7HF, UK.

[b] School of Chemistry, University of Leeds, Woodhouse Lane, Leeds, LS2 9JT, UK.

E-mail: Y.Ning@sheffield.ac.uk; s.p.armes@sheffield.ac.uk

Additional Experimental and Characterization

Preparation of gold nanoparticles dispersed in isohexadecane and magnetite nanoparticles dispersed in isohexadecane. The dispersion of gold nanoparticles in isohexadecane (≈ 5 mg mL^{-1}) was prepared as follows. $HAuCl_4 \cdot 3H_2O$ (50 mg) was weighed into a 50 mL round-bottomed flask, followed by addition of oleylamine (10 mL) and oleylamine (10 mL). The resulting solution was immersed in an oil bath at 120 °C for 1 h. The gold nanoparticles (stabilized by oleylamine, ~10 nm) were centrifuged three times at 15,000 rpm for 20 min with successive supernatants being replaced with ethanol and finally redispersed in isohexadecane with the aid of an ultrasonic bath.

The dispersion of magnetite nanoparticles in isohexadecane (~10 nm) was prepared as follows. $FeCl_3 \cdot 6H_2O$ (6.08 g) and $FeCl_2 \cdot 4H_2O$ (2.98 g) were added to a 100 mL round-bottomed flask equipped with a stirrer bar and dissolved in water (50 mL). This aqueous solution was heated up to 90 °C and 35% ammonia solution (15 mL) and oleic acid (0.989 g) were rapidly added with continuous stirring and the reaction was allowed to continue for 2 h at 90 °C. The resulting magnetite particles were centrifuged three times at 10,000 rpm for 10 min with successive supernatants being replaced with water and then centrifuged twice at 15,000 rpm for 20 min with successive supernatants being replaced with ethanol. Finally, the magnetite nanoparticles were redispersed into either isohexadecane with the aid of an ultrasonic bath.

Raman spectroscopy. Raman spectra were recorded using a Renishaw 2000 Raman microscope equipped with a 785 nm diode laser at a resolution of 2 cm^{-1}. Spectra were averaged over 256 scans.

Powder X-ray diffraction (XRD). Powder XRD measurements were made using a Bruker D2 Phaser Desktop X-ray diffractometer equipped with Ni-filtered Cu Kα radiation (λ = 1.542 Å) operating at an accelerating voltage and emission current of 30 kV and 10 mA, respectively.

Analytical Centrifugation (LUMiSizer). Nanoemulsion size distributions were assessed using a LUMiSizer analytical photocentrifuge (LUM GmbH, Berlin, Germany) at 20 °C. Measurements were

conducted on diluted nanoemulsions (2.0% v/v) using 2 mm path length polyamide cells at 2,000 rpm for 1,000 profiles (allowing 10 s between profiles).

Thermogravimetric analysis (TGA). TGA was conducted using a Perkin-Elmer Pyris 1 TGA instrument, heating from 20 °C to 900 °C under air at a heating rate of 10 °C per min. In principle, complete thermal decomposition of $CaCO_3$ should give 56% CaO and 44% CO_2 by mass. This was verified for pure calcite in a control experiment as shown in Fig. S8. The occlusion of nanoemulsion droplets within calcite crystals should lead to a reduction in the CaO content (see Fig. S8). Given that both methyl myristate and $PMAA_{156}$-$PLMA_y$ diblock copolymer are completely pyrolyzed at 550 °C (see Fig. S8), any remaining residues can be assigned to CaO.

Based on the experimental % CaO (P_{CaO}), the % CO_2 can be calculated to be:

$$P_{CO_2} = \frac{44 \times P_{CaO}}{56}$$

So the extent of nanoemulsion occlusion $P_{nanoemulsion}$ by mass can be calculated using the following equation:

$$P_{nanoemulsion}(by\ mass) = 100 - P_{CaO} - \frac{44 \times P_{CaO}}{56} = 100 \times (1 - \frac{P_{CaO}}{56})$$

Given the densities of methyl myristate (ρ_m) and calcite (ρ_c), it follows that:

$$P_{nanoemulsion}(by\ volume) = \frac{100 \times (56 - P_{CaO}) \times \rho_c}{(56 - P_{CaO}) \times \rho_c + P_{CaO} \times \rho_m}$$

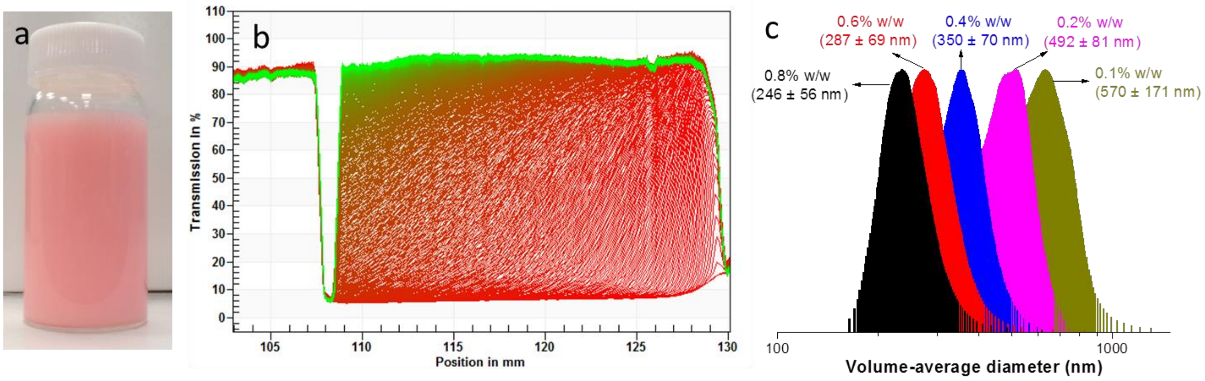

Fig. S1 (a) Digital photograph of a 492 ± 81 nm diameter methyl myristate-in-water nanoemulsion; (b) typical transmission profiles obtained during analytical centrifugation of this methyl myristate-in-water nanoemulsion at 2,000 rpm (corresponding to 510g) using a LUMiSizer (1,000 profiles, recorded at ten-second intervals); (c) representative droplet size distributions determined for five nanoemulsions prepared using various M_{156}-L_{80} copolymer concentrations ranging from 0.1% w/w to 0.8% w/w (assuming a droplet density of 0.855 g cm^{-3} in each case).

Given that the density of methyl myristate (0.855 g cm^{-3}) is less than that of water, methyl myristate-in-water nanoemulsion droplets cream (rather than sediment) when subjected to centrifugation. The LUMiSizer is a commercial analytical centrifugation instrument that employs proprietary STEP™ Technology (Space- and Time-resolved Extinction Profiles), which allows the measurement of the intensity of transmitted near-infrared light as a function of time and position over the entire length of the sample cell simultaneously. The gradual progression of these transmission profiles, as shown in **Fig. S1b**, contains information on the rate of creaming and therefore enables assessment of the nanoemulsion droplet size distribution.[1-3]

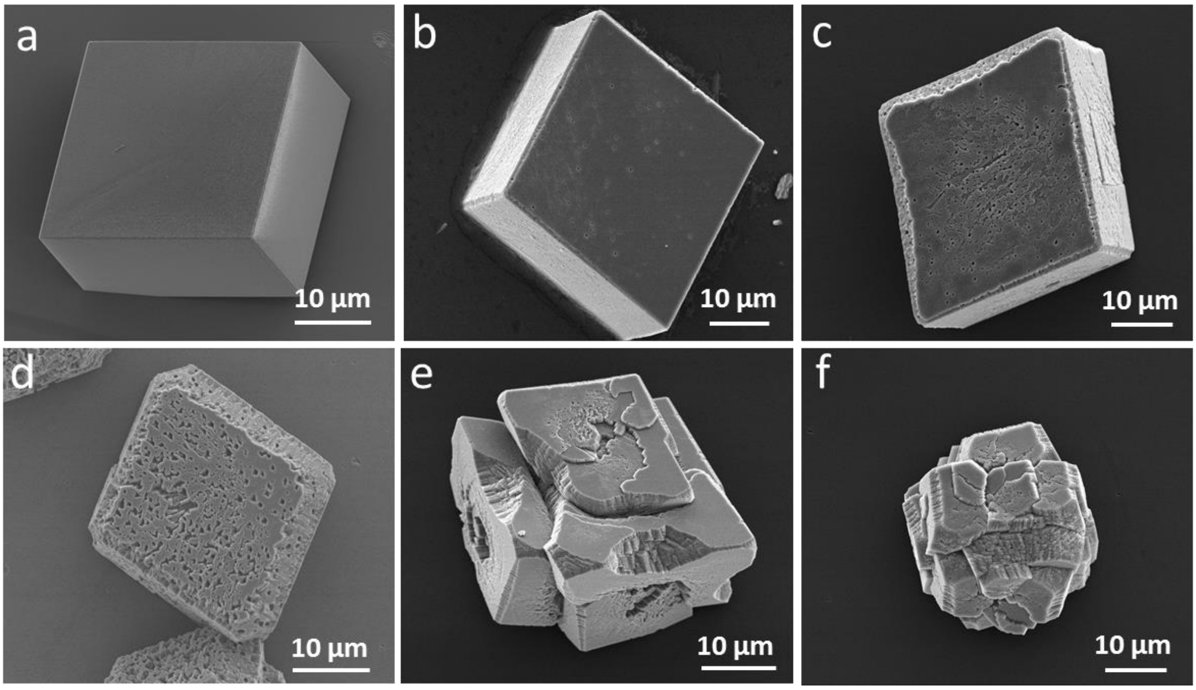

Fig. S2 Representative SEM images obtained for $CaCO_3$ crystals prepared in the presence of $PMAA_{156}$-$PLMA_{80}$ stabilized methyl myristate-in-water nanoemulsions (each prepared using 0.2% w/w $PMAA_{156}$-$PLMA_{80}$) at the following nanoemulsion concentrations: (a) 0% v/v (control calcite); (b) 0.01% v/v; (c) 0.05% v/v; (d) 0.10% v/v; (e) 0.20% v/v; (f) 0.50% v/v. Well-defined rhombohedral $CaCO_3$ particles were obtained at nanoemulsion concentrations of 0.01% v/v to 0.10% v/v, but truncated edges became more pronounced at higher nanoemulsion concentrations. In particular, using a nanoemulsion concentration ≥ 0.20% v/v produced only ill-defined polycrystalline crystals, see SEM images shown in (e-f).

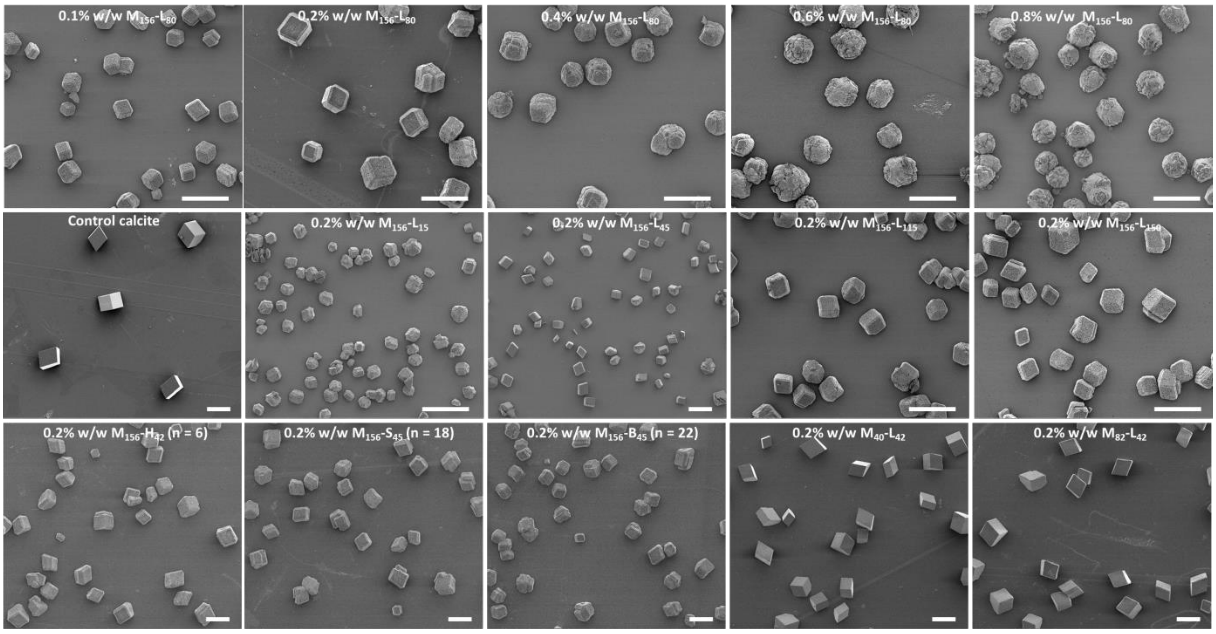

Fig. S3 SEM images recorded for calcite crystals precipitated in the absence or presence of nanoemulsion droplets stabilized by various anionic amphiphilic diblock copolymers (see Scheme 1 for the relevant chemical structures). All scale bars equal 50 µm.

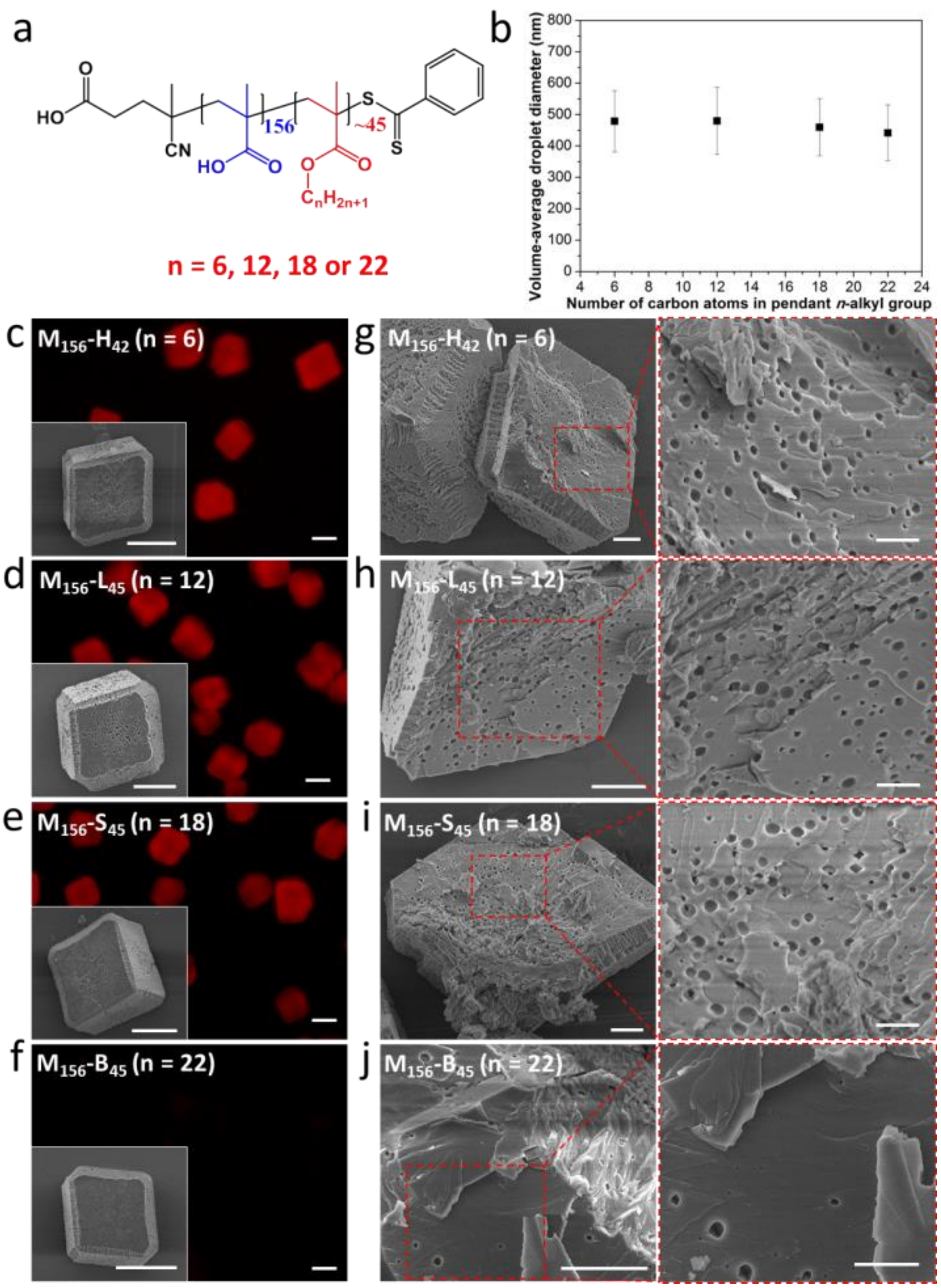

Fig. S4 Effect of varying the *n*-alkyl group on the hydrophobic block of the diblock copolymer emulsifier on the extent of occlusion of oil droplets within $CaCO_3$. (a) Chemical structure of $PMAA_x$-based diblock copolymers with varying *n*-alkyl groups on the hydrophobic poly(*n*-alkyl methacrylate) block; (b) mean droplet diameter of methyl myristate-in-water nanoemulsions obtained for various diblock copolymer emulsifiers. (c)-(j) $CaCO_3$ particles precipitated in the presence of various nanoemulsions prepared using the same *molar* concentration (2.94×10^{-7} M) of a series of diblock copolymer emulsifiers. (c-f) Fluorescence microscopy images; corresponding SEM images (see insets) illustrating the surface morphology of the intact $CaCO_3$ particles. More SEM images are provided in Fig. S3. (g-j) SEM images revealing the internal morphology of randomly-fractured $CaCO_3$ particles. Scale bars for the fluorescence microscopy images, inset SEM images, low magnification SEM images and high magnification SEM images are 20 µm, 10 µm, 5 µm and 2 µm, respectively.

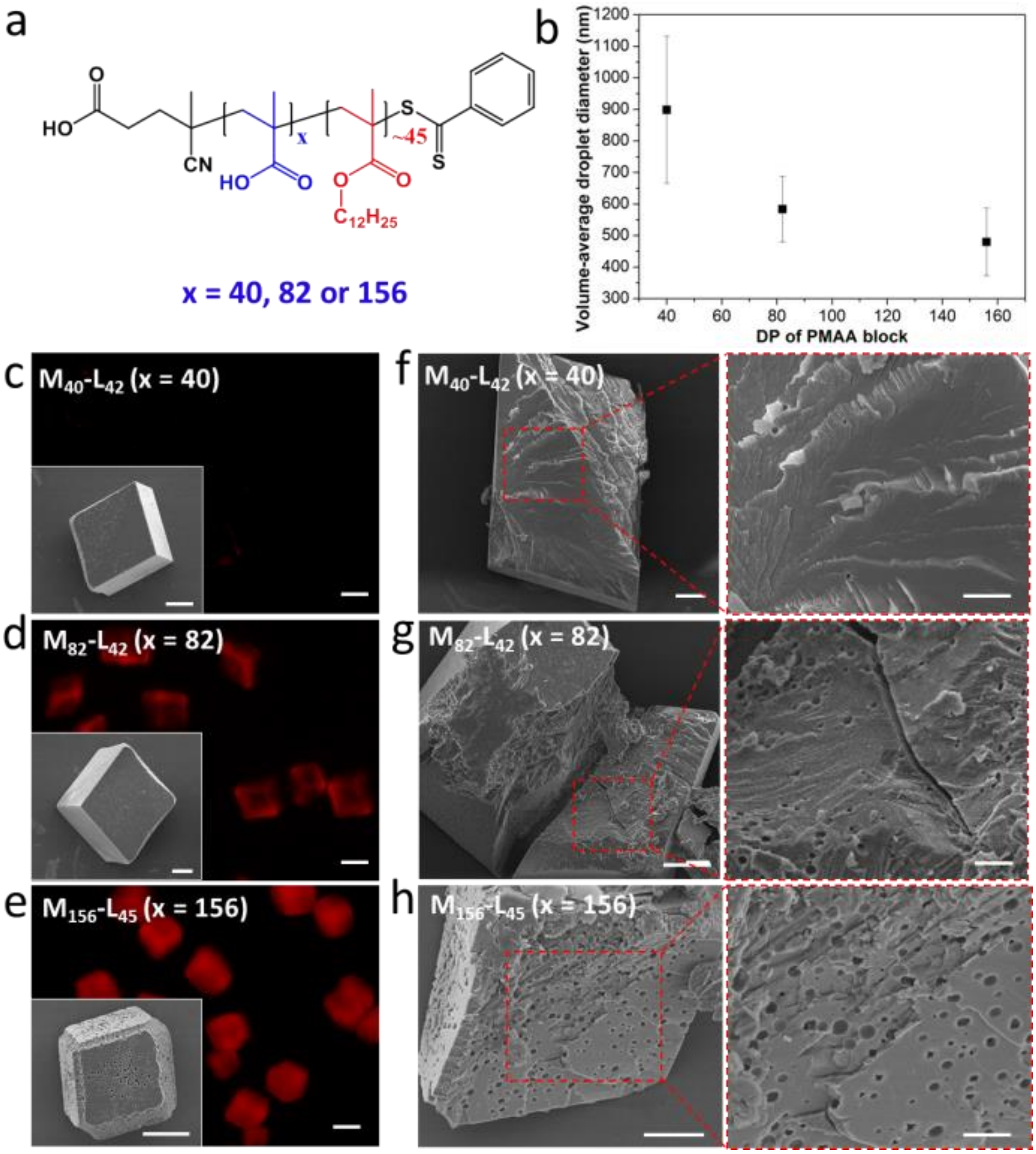

Fig. S5 Effect of varying the PMAA$_x$-PLMA$_{\sim45}$ copolymer composition on the extent of occlusion of oil droplets within calcite. (a) Chemical structure of PMAA$_x$-PLMA$_{\sim45}$ diblock copolymer; (b) reduction in the mean droplet diameter of methyl myristate-in-water nanoemulsions on increasing the PMAA block DP of the PMAA$_x$-PLMA$_{\sim45}$ emulsifier. (c)-(h) $CaCO_3$ particles precipitated in the presence of various nanoemulsions stabilized using the same *molar* concentration of PMAA$_x$-PLMA$_{\sim45}$ (2.94×10^{-7} M), where x = 40, 82 or 156. (c-e) Fluorescence microscopy images; corresponding SEM images (see insets) illustrating the surface morphology of the intact $CaCO_3$ particles. More SEM images are provided in Fig. S3. (f-h) SEM images revealing the internal morphology of randomly-fractured $CaCO_3$ particles. Scale bars for the fluorescence microscopy images, inset SEM images, low magnification SEM images and high magnification SEM images are 20 µm, 10 µm, 5 µm and 2 µm, respectively.

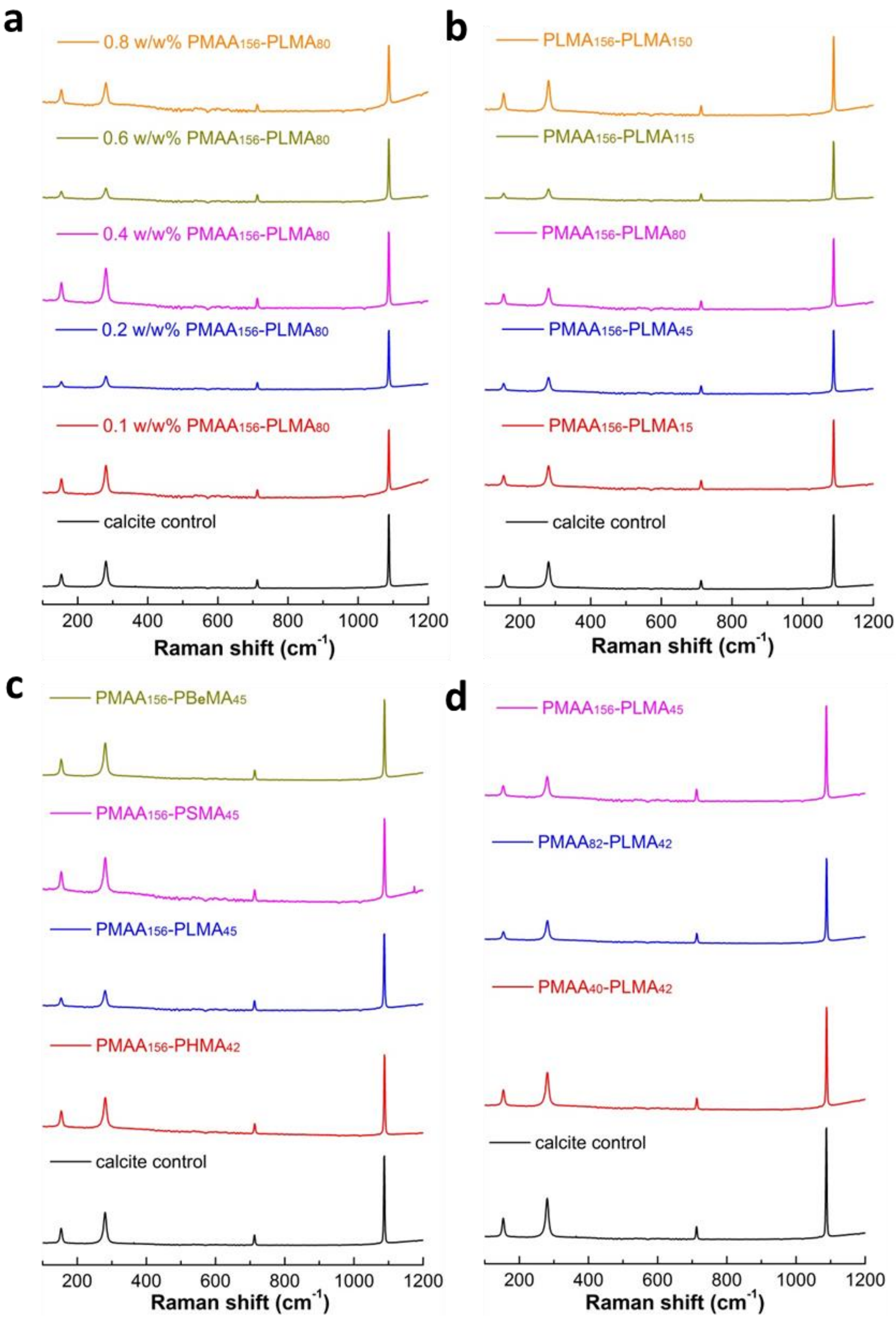

Fig. S6 Raman spectra recorded for a pure calcite control and calcium carbonate precipitated in the presence of methyl myristate-in-water nanoemulsions stabilized using various diblock copolymers (see labels for details). Characteristic Raman bands for calcite were detected at 153 and 281 cm^{-1} (lattice modes), 712 cm^{-1} (υ_4) and 1088 cm^{-1} (υ_1) for both the control and the nanoemulsion-loaded nanocomposite crystals.

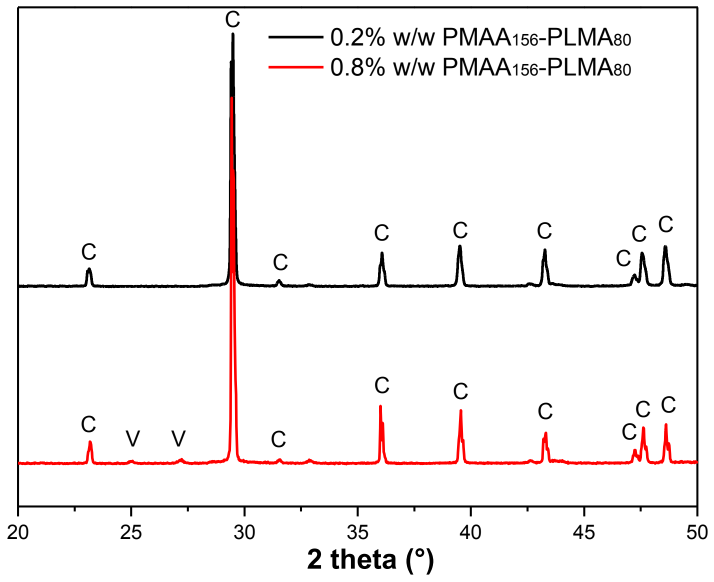

Fig. S7 Powder X-ray diffractograms obtained for calcite (*c*) crystals precipitated in the presence of methyl myristate-in-water nanoemulsions stabilized using either 0.2% w/w or 0.8% w/w $PMAA_{156}$-$PLMA_{80}$. A small amount of vaterite (*v*) is also observed at the higher copolymer concentration.

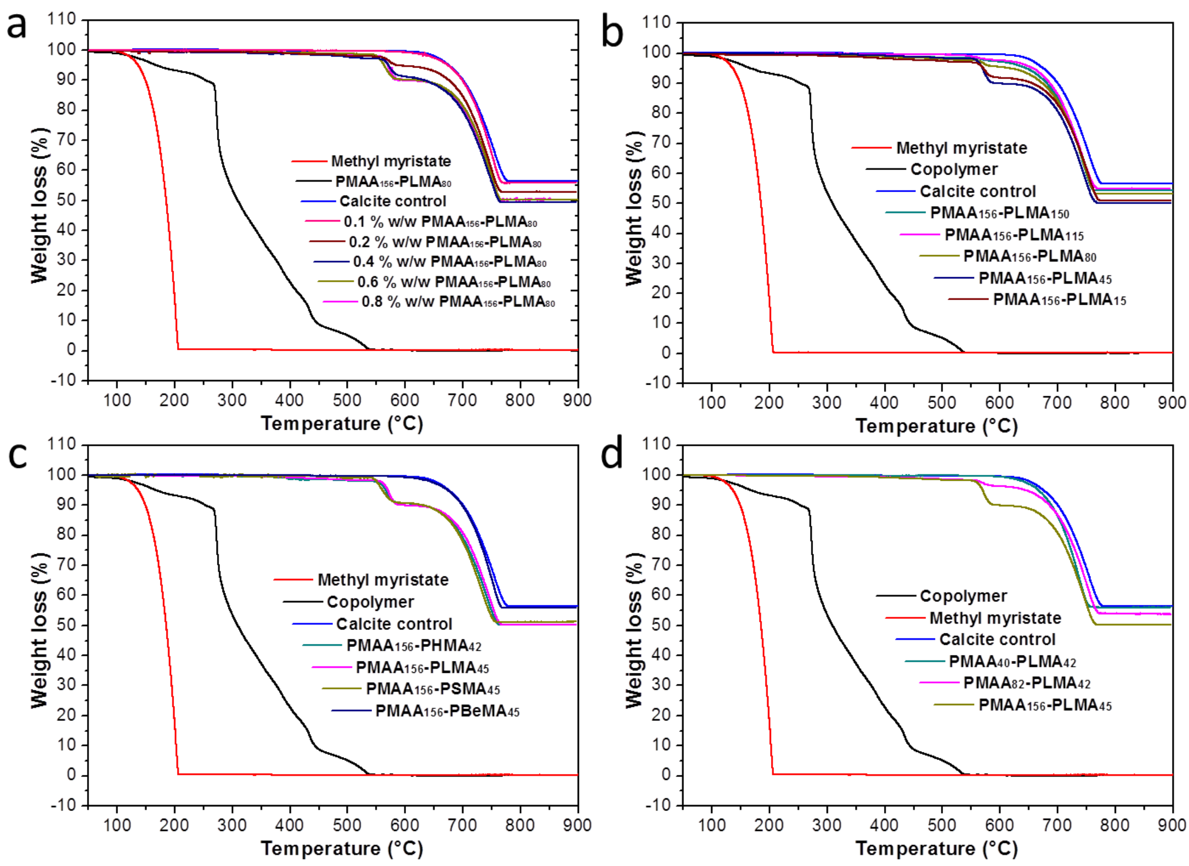

Fig. S8 Thermogravimetric analysis (TGA) curves recorded for methyl myristate, diblock copolymer nanoparticles alone, and calcite crystals precipitated in the absence or presence of methyl myristate-in-water nanoemulsions stabilized using (a) $PMAA_{156}$-$PLMA_{80}$ diblock copolymer at various concentrations; (b) a series of five $PMAA_{156}$-$PLMA_{y}$ diblock copolymers (where = 15 to 150) at fixed molar concentration of 2.94×10^{-7} M; (c) a series of diblock copolymers (where the core-forming monomer is either HMA, LMA, SMA, or BeMA) at a fixed molar concentration of 2.94×10^{-7} M, (d) a series of three $PMAA_{x}$-$PLMA_{\sim 45}$ diblock copolymers (where x = 40, 82 or 156) at a fixed molar concentration of 2.94×10^{-7} M.

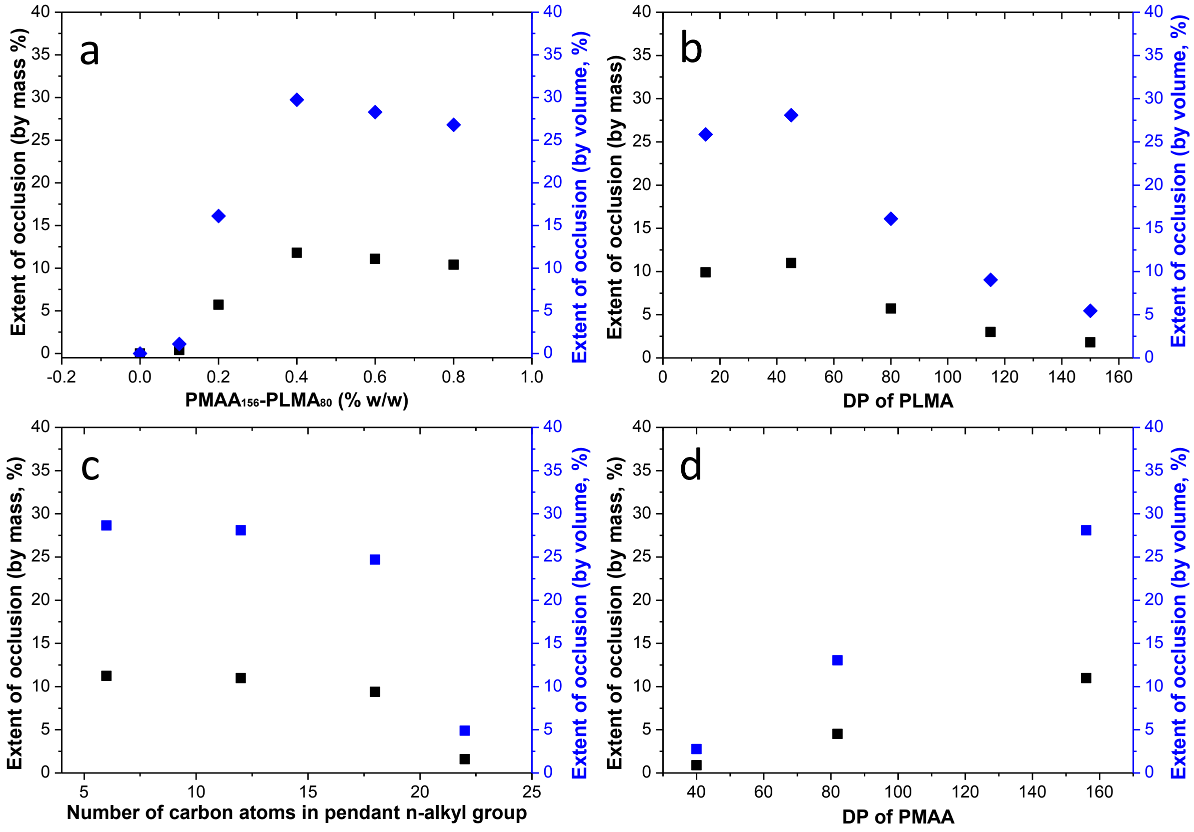

Fig. S9 Extent of occlusion (by mass and by volume) determined by thermogravimetric analysis (TGA). (a) Effect of varying the $PMAA_{156}$-$PLMA_{80}$ concentration; (b) Effect of varying the DP (y) of the PLMA block for a series of five $PMAA_{156}$-$PLMA_{y}$ diblock copolymers; (c) effect of varying the pendant *n*-alkyl group in the hydrophobic block for a series of four $PMAA_{156}$-based diblock copolymers; (d) effect of varying the DP of the hydrophilic $PMAA_{x}$ block for a series of three $PMAA_{x}$-$PLMA_{\sim 45}$ diblock copolymers.

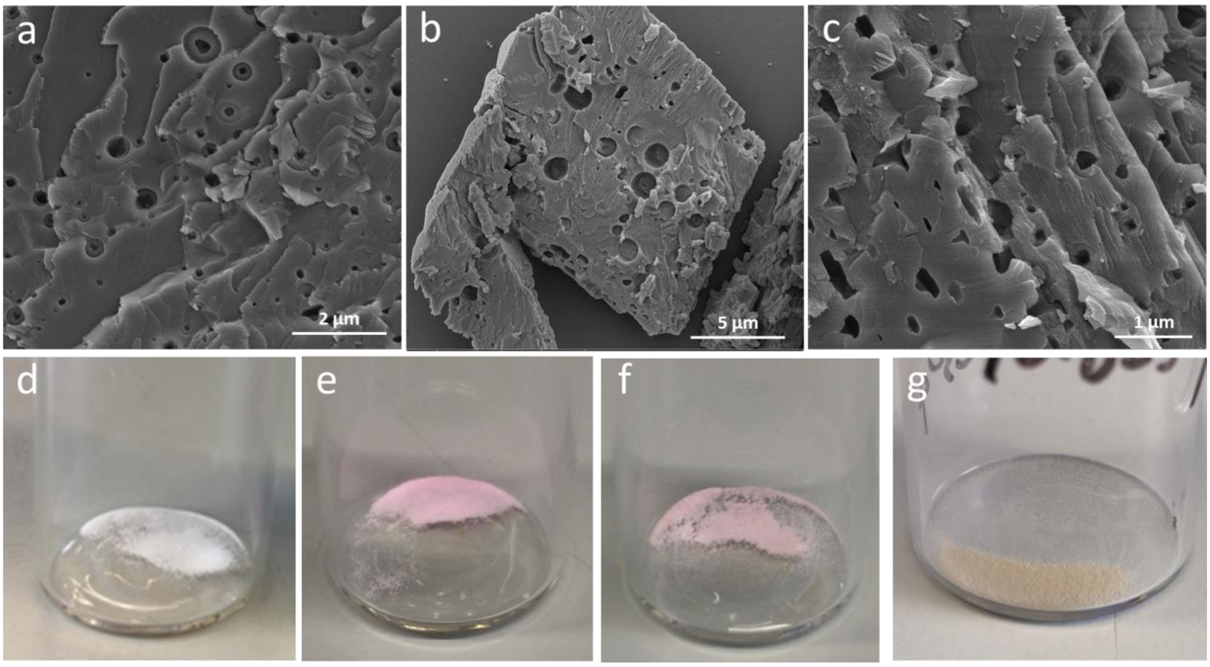

Fig. S10 SEM images recorded for calcite crystals precipitated in the presence of (a) sunflower oil-in-water nanoemulsions, (b) multi-component fragrance-in-water nanoemulsions and (c) isohexadecane-in-water nanoemulsions. Digital photographs recorded for (d) control calcite crystals, (e) calcite crystals occluded with isohexadecane-in-water nanoemulsions with ~10 nm diameter gold nanoparticles dispersed within the oil droplets (gold nanoparticle concentration ≈ 5 mg mL^{-1}), (f) the same gold nanoparticle-loaded calcite crystals after heating in air up to 900 °C (TGA analysis) and (g) calcite crystals occluded with isohexadecane-in-water nanoemulsions with ~10 nm diameter Fe_3O_4 nanoparticles dispersed within the oil droplets. Incorporation of the gold nanoparticles conferred a pinkish-red colour on the calcite crystals, which is retained after thermal annealing.

Table S1. Summary of synthesis details of $PMAA_{156}$, $PMAA_{82}$ and $PMAA_{40}$ macro-CTAs.

macro-CTA	Monomer (MAA)		RAFT agent (CPCP)		Initiator (ACVA)		[CTA]/[Initiator]	Target DP	Solvent (ethanol)	MAA conversion (%)	GPC	
	Mass (g)	Moles (mol)	Mass (g)	Moles (mmol)	Mass (g)	Moles (mmol)			Mass (g)		M_n /g mol^{-1}	M_w/M_n
**$PMAA_{156}$**	**10.0**	**0.116**	**0.162**	**0.581**	**0.033**	**0.116**	**5.0**	**200**	**15.0**	**89**	**17,400**	**1.10**
**$PMAA_{82}$**	**10.0**	**0.116**	**0.271**	**0.968**	**0.054**	**0.194**	**5.0**	**120**	**15.0**	**80**	**9,200**	**1.15**
**$PMAA_{40}$**	**10.0**	**0.116**	**0.649**	**2.324**	**0.130**	**0.465**	**5.0**	**50**	**15.0**	**77**	**4,800**	**1.15**

Table S2. Summary of the number-average molecular weight (M_n) and dispersity (M_w/M_n) data obtained for various diblock copolymers, nanoemulsion diameter and the extents of occlusion for the corresponding nanoemulsions within $CaCO_3$.

Copolymer ID	GPC data		Nanoemulsion		Extent of nanoemulsion occlusion	
	M_n (g mol^{-1})	M_w/M_n	Copolymer concentration	Nanoemulsion diameter (nm)	By mass (%)	By volume (%)
$PMAA_{156}$-$PLMA_{80}$	31,600	1.25	0.1% w/w	570 ± 171	0.40	1.12
$PMAA_{156}$-$PLMA_{80}$	31,600	1.25	0.2% w/w	492 ± 81	5.70	16.11
$PMAA_{156}$-$PLMA_{80}$	31,600	1.25	0.4% w/w	350 ± 70	11.80	29.75
$PMAA_{156}$-$PLMA_{80}$	31,600	1.25	0.6% w/w	287 ± 79	11.1	28.30
$PMAA_{156}$-$PLMA_{80}$	31,600	1.25	0.8% w/w	246 ± 56	10.4	26.80
$PMAA_{156}$-$PLMA_{15}$	21,000	1.18	2.94×10^{-7} M	462 ± 104	9.91	25.86
$PMAA_{156}$-$PLMA_{45}$	26,500	1.25	2.94×10^{-7} M	480 ± 107	10.97	28.09
$PMAA_{156}$-$PLMA_{80}$	31,600	1.25	2.94×10^{-7} M	492 ± 81	5.70	16.11
$PMAA_{156}$-$PLMA_{115}$	38,500	1.23	2.94×10^{-7} M	507 ± 102	3.00	9.03
$PMAA_{156}$-$PLMA_{150}$	44,100	1.28	2.94×10^{-7} M	527 ± 104	1.80	5.45
$PMAA_{40}$-$PLMA_{42}$	13,900	1.15	2.94×10^{-7} M	899 ± 233	0.88	2.75
$PMAA_{82}$-$PLMA_{42}$	17,100	1.14	2.94×10^{-7} M	584 ± 104	4.51	13.03
$PMAA_{156}$-$PHMA_{42}$	21,400	1.12	2.94×10^{-7} M	479 ± 98	11.23	28.64
$PMAA_{156}$-$PSMA_{45}$	27,200	1.18	2.94×10^{-7} M	460 ± 91	9.38	24.70
$PMAA_{156}$-$PBeMA_{45}$	28,800	1.18	2.94×10^{-7} M	442 ± 89	1.59	4.88

N.B. The carboxylic acid groups on the PMAA chains were fully methylated using excess trimethylsilyldiazomethane. Molecular weights were calculated relative to a series of near-monodisperse poly(methyl methacrylate) standards.

References

1. T. Detloff, T. Sobisch and D. Lerche, *Powder Technol.*, 2007, 174, 50-55.
2. T. Detloff, T. Sobisch and D. Lerche, *Part. & Part. Syst. Char.*, 2006, 23, 184-187.
3. K. L. Thompson, N. Cinotti, E. R. Jones, C. J. Mable, P. W. Fowler and S. P. Armes, *Langmuir*, 2017, 33, 12616-12623.
